# Self-reported prescription medication use and polypharmacy following physical combat injury in the UK: the ADVANCE cohort study

**DOI:** 10.3389/fphar.2026.1893711

**Published:** 2026-10-01

**Authors:** Daniel Dyball, Laura E. Grover, Susie Schofield, Sarah Dixon Smith, Alex Kumar, Alexander N. Bennett, Christopher J. Boos, Anthony M. J. Bull, Neil S. N. Graham, Cheryl R. Z. See, Lydia Orton, Grace Blissitt, David J. Sharp, Nicola T. Fear, Harriet I. Kemp

**Affiliations:** 1 King’s Centre for Military Health Research, King’s College London, London, United Kingdom; 2 Department of Bioengineering, Imperial College London, London, United Kingdom; 3 Pain Research Group, Faculty of Medicine, Imperial College London, London, United Kingdom; 4 Academic Department of Military Anaesthesia and Pain Management, Research & Clinical Innovation (RCI), Birmingham, United Kingdom; 5 Academic Department of Military Rehabilitation, Defence Medical Rehabilitation Centre, Birmingham, United Kingdom; 6 Centre for Injury Studies, Department of Bioengineering, Imperial College London, London, United Kingdom; 7 Faculty of Health & Social Sciences, Bournemouth University, Poole, United Kingdom; 8 Department of Brain Sciences, Imperial College London, London, United Kingdom; 9 UK Dementia Research Institute Centre for Care Research and Technology, Imperial College London, London, United Kingdom; 10 Academic Department of Military Mental Health, King’s College London, London, United Kingdom; 11 Department of Surgery and Cancer, Imperial College London, London, United Kingdom

**Keywords:** ADVANCE cohort, military personnel, polypharmacy, prescription drugs, wounds and injuries

## Abstract

**Introduction:**

The consequences of serious physical traumatic injury may require pharmacological intervention, however long-term use and polypharmacy can increase risk of morbidity/mortality. This study aims to describe prescription medication use in a cohort of UK Armed Forces personnel who sustained physical combat injuries during the Afghanistan conflict (injured group) compared to demographically similar personnel without such injuries (uninjured group) a median of 11 years post injury.

**Methods:**

One thousand and fifty one participants of the ADVANCE cohort (injured group *n* = 526 uninjured group *n* = 525), including individuals with (n = 141) and without (*n* = 384) limb loss or with (n = 226) and without (*n* = 300) deployment-related Traumatic Brain Injury (TBI), reported medication use during a clinical interview. Overall incidence of prescribing any medication, specific anatomical/therapeutic classes (e.g., Central Nervous System (CNS), psychotropic or analgesic) and medications with pain or mental health indications were investigated. General (≥5 medications), CNS (≥5), psychotropic (≥2) and analgesic (≥2) polypharmacy was also investigated.

**Results:**

Compared to the uninjured group, the injured group reported a greater prevalence of prescription medication use (17.8% vs. 29.7%), including medications acting on the CNS (8.0% vs. 17.2%), psychotropics (6.3% vs. 11.8%) and analgesics (2.2% vs. 8.6%). Prevalence of polypharmacy was very low in the cohort (0%–4.4%). Incidence Rate Ratios (IRR) and 95% confidence intervals indicated that injured personnel were currently using on average over twice as many prescription medications as those in the uninjured group (IRR 2.15 (1.66, 2.92)), including medications acting on the CNS (IRR 2.67 (1.77, 4.08)), and psychotropics (IRR 2.03 (1.27, 3.25)). Similar elevations were present in those injured with and without limb loss and especially elevated in those with deployment-related TBI. Use of psychotropics amongst personnel injured with limb loss were predominantly for non-mental health indications (e.g., sleep, pain).

**Conclusion:**

Pharmacological intervention is not uncommon amongst UK Armed Forces personnel who sustained serious physical injuries, but the prevalence of polypharmacy is low. Effective prescribing practices should ensure optimal recovery whilst minimising the risk of adverse events.

## 1 Introduction

Injuries sustained by UK Armed Forces personnel during deployments to Afghanistan were associated with complex polytrauma, with a greater number of personnel surviving severe injuries than in previous deployments to Iraq or Northern Ireland (McGuire et al., 2019). As a result of these injury profiles and complex sequalae of physical and mental health issues, multiple pharmacological intervention might be required (Melcer et al., 2020). When a high number of medications are prescribed to an individual, this is known as polypharmacy (Masnoon et al., 2017). Whilst perhaps necessary in the face of complex multimorbidity (Sciarra et al., 2019), polypharmacy must be carefully managed as the risk of adverse events increases the more medications are administered (Eide and Stahlman, 2018).

Whilst criteria exist for identifying potentially inappropriate medications for older patients with multimorbidity (e.g., Beers criteria), no such criteria exists for younger individuals with post-injury traumatic sequalae (Fick et al., 2003). Research is also limited by problems with heterogeneity in definitions of polypharmacy (Masnoon et al., 2017), and unique patterns in prescription medication can be observed depending on the definition of polypharmacy that is adopted. In this paper, we adopt the definitions of polypharmacy most utilised in the literature: general polypharmacy (≥5 medications); psychotropic polypharmacy (≥2 psychotropic medications); Central Nervous System (CNS) polypharmacy (≥5 medications acting on the CNS) and analgesic polypharmacy (≥2 analgesic medications) (Masnoon et al., 2017; Rothschild, 2021; Raut et al., 2025; Collett et al., 2016).

Polypharmacy is primarily investigated in older adults. In this population, general polypharmacy has been observed to be associated with increased mortality risk (Li et al., 2022). Limited research is available examining polypharmacy in younger groups, though increased risk of mortality has been observed in US middle aged veterans with general polypharmacy (Guillot et al., 2024). Some shared mechanisms of mortality risk through polypharmacy between younger and older adults may be through increased risk of adverse drug events/drug-drug interactions, and pharmacokinetics/pharmacodynamics depending on disease severity, progression and time on medication (Li et al., 2022). Additionally, increased suicide-related behaviour and drug/alcohol overdose has been observed in US military personnel who deployed to Iraq/Afghanistan with CNS polypharmacy (Collett et al., 2016), alongside associations with depression, PTSD and comorbid Traumatic Brain Injury (TBI). Previous research on Australian Vietnam veterans with PTSD found that 33.1% were treated with psychotropic polypharmacy (Theal et al., 2020). This polypharmacy was associated with greater PTSD symptom severity, depression and suicidality. Australian veterans admitted to a mental health facility with PTSD were also found to have very high prevalence of both general polypharmacy (76.7%) and psychotropic polypharmacy (75.3%) (Mellor et al., 2022). In a recent meta-analysis, general polypharmacy and psychotropic polypharmacy were estimated to be 49% and 36% respectively amongst military and veteran populations (Raut et al., 2025). However, the majority of this data was based on US research, and there was considerable heterogeneity in estimates which were not explained by age, geographical location or medication threshold definitions. It is likely that certain experiences during military service, such as combat injury, might affect these estimates and in the case of polytrauma, multiple, differing guidelines for individual conditions (e.g., TBI, pain, post-traumatic stress disorder) are thought to be partially responsible for polypharmacy approaches to prescribing (Brown-Taylor et al., 2021). No known research has investigated this within UK Armed Forces personnel.

Polypharmacy is common in those living with chronic pain. Whilst combined therapy, using medications from multiple drug classes to target different physiological mechanisms, may improve pain management (Chaparro et al., 2012), it also increases the potential for adverse effects (Zahlan et al., 2023; De Clifford-Faugère et al., 2025). There is an increasing awareness that combinations such as opioids and gabapentinoids are associated with increased harm, including risk of death (Kalk et al., 2022; Torrance et al., 2020; Muller et al., 2024). In US veteran populations historically opioid prescribing rates in those with chronic pain have been high (Lakshman et al., 2024), but changes in approach to pain management has led to a considerable reduction (Jannace et al., 2020; Goldman et al., 2022). However, it is unclear how common co-prescribing of gabapentinoids and opioids is in military populations.

When investigating prescription medication use, it is important to investigate not only therapeutic classifications of medications, but also the indications for why those medications are being used. For example, studies have described how antidepressant medications are also prescribed for broader, frequently off-label, indications (e.g., sleep and pain) (Gardarsdottir et al., 2007; Aarts et al., 2016).

### 1.1 Aims

This study aims to describe prescription medication use and polypharmacy in a cohort of UK Armed Forces personnel who sustained physical combat injuries compared to a demographically similar group who sustained no such injuries. Injury profiles will be investigated, specifically injuries with/without associated limb loss and with/without deployment-related TBI. We will investigate overall prevalence of prescription medications, anatomical/therapeutic classes of medications including CNS, psychotropics and analgesics as well as medications with self-reported indications of mental health or pain. We also aim to report on the use of opioids, gabapentinoids and any co-prescription of these two classes of medication.

### 1.2 Hypothesis

Male UK Armed Forces personnel who sustained physical combat injuries, including individuals with/without associated limb loss or deployment-related TBI, will be prescribed a greater number of medications compared to demographically similar UK Armed Forces personnel who sustained no such injuries.

## 2 Methods

### 2.1 Study design and participants

Full details of the ADVANCE cohort protocol can be found elsewhere (Bennett et al., 2020). The baseline cohort consists of an injured group (n = 579), who were recruited from a sample of UK Armed Forces personnel who deployed to Afghanistan (Operation Herrick) between 2003–2014 and sustained physical combat injuries requiring aeromedical evacuation from theatre to a UK hospital, and an uninjured group (n = 566), who were recruited from individuals who were frequency-matched at sampling to the injured group based on their age, rank, regiment, deployment era and role on deployment. Baseline recruitment attained a 59.6% response rate for eligible injured group participants, which represented 55.1% of eligible participants with a very serious or serious NOTICAS classification (Dyball et al., 2022). This analysis reports on the first follow up data from the ADVANCE cohort, collected a median of 11 years following injury/deployment of interest (Dyball et al., 2025) and retaining 92.0% of the baseline sample (Dyball et al., 2025).

### 2.2 Procedure

Participants were contacted to participate in the first follow up appointment for ADVANCE through postal, email and telephone invitations, having previously consented to take part in follow up assessments at baseline. The study day took place at the Defence Medical Rehabilitation Centre (Stanford Hall) and included medical assessments, a clinical interview led by a research nurse and a self-report confidential questionnaire.

### 2.3 Ethics

The ADVANCE Study has approval from the Ministry of Defence Research Ethics Committee (MODREC; protocol No:357/PPE/12). All participants gave written informed consent.

### 2.4 Measures

#### 2.4.1 Dependent variables

##### 2.4.1.1 Prescription medications

Participants were asked to bring all medications they were currently using (e.g., packaging, general information) to discuss during the clinical interview with a research nurse. Participants indicated whether each medication was ‘over the counter’ or ‘prescription’. Only medications identified as prescription are analysed in this study. Medications reported as over the counter were reviewed and re-classified as prescription medication if the medication was only available as a prescription medication in the UK. Medications classified as over the counter that could have been obtained illicitly (e.g., steroids) were not considered to be recoded as prescription medications (n = 2). Medications were coded using the Anatomical Therapeutic Chemical Classification Index (ATC) 2020 (World Health Organisation Collaborating Centre for Drug Statistics Methodology, 2020).

CNS medications were categorised based on the medications in the ATC framework “Central Nervous System” anatomical class, psychotropic medications were categorised based on therapeutic classifications of ‘Psycholeptic’ or ‘Psychoanaleptic’ classes, and analgesic medications were categorised on therapeutic classification of ‘Analgesic’. These data were coded as count data referring to the number of medications that person was currently on within each relevant class. Prescriptions of any gabapentin/pregabalin analgesic medications and prescriptions of gabapentin/pregabalin medication alongside other opioid medication were coded separately.

Participants were asked about the indication of each medication, which was written as free text by the research nurse during the interview. Authors DD, HIK and LEG coded indication-categories from this free text, and agreement was sought for all categories between the authors. The two primary indications for this study include ‘pain’ and ‘mental health’.

##### 2.4.1.2 Polypharmacy

General polypharmacy was categorised as ‘on ≥five prescription medications’, CNS polypharmacy as ‘on ≥five CNS prescription medications’, psychotropic polypharmacy as ‘on ≥two prescription psychotropic medications’ and analgesic polypharmacy as ‘on ≥two prescription analgesic medications’.

#### 2.4.2 Independent variables

##### 2.4.2.1 Combat-injury

Information on combat-injury was collected from electronic medical records and supplemented by self-report in the clinical interview. Injury groups were coded as a binary variable identifying the uninjured group and the injured group. Injury profiles were coded based on presence of limb loss or deployment-related TBI. Participants with isolated partial amputations (toe/partial foot/finger/partial hand) without limb loss were coded as injuries without limb loss. Deployment related TBI was established through the use of the Ohio-State University Traumatic Brain injury Identification Method (Corrigan and Bogner, 2007), review of Joint Theatre Trauma Register records and past medical history alongside assessment of clinical magnetic resonance imaging scans by a consultant neuroradiologist (Blissett et al., 2026). A deployment-related TBI was categorised as any evidence of a mild (probable) or moderate-severe (definite) TBI, in line with Mayo classifications (Ma et al., 2007).

##### 2.4.2.2 Socioeconomic status

Rank at the point of sampling was used as a proxy for socioeconomic status (Yoong et al., 1999). Participants were coded as other rank/junior non-commissioned officer (NATO rank OR2-OR4), senior non-commissioned officer (OR5-OR9) and officer rank (OF1-OF9).

##### 2.4.2.3 Age

Age at follow up assessment (in years) was used.

##### 2.4.2.4 Ethnicity

Ethnicity was self-reported during the clinical interview. Answers were coded as “Asian”, “Black”, “Mixed” or “White”. A binary code was derived from this data to represent “White” and “Ethnic minorities (not including white minorities)”.

##### 2.4.2.5 Time since injury/deployment of interest

Time since injury/deployment of interest was calculated by the difference between participants age at index injury for injured personnel or age at sampled deployment+0.5 (to reflect their average age during sampled year) for the uninjured group and participants age at follow-up assessment.

### 2.5 Data analysis

Data analysis utilised STATA Now 19. All models controlled for rank at sampling and age at follow up assessment (Thomas et al., 2016; Hudson, 2005). A longitudinal weight for the first follow-up was calculated by multiplying an attrition weight at the first follow up by the overall baseline weight (Dyball et al., 2025). Weighted percentages are presented along with unweighted cell counts in demographic tables. Cell counts of n < 5 are not reported in line with UK Defence Statistics rounding policy.

To investigate prescription medication use, generalised linear models with negative binomial distribution were utilised to account for overdispersion (assessed via goodness of fit following a Poisson model). The dependent variables were count data, indicating the number of a) any prescription medications b) medications within therapeutic classes (e.g., CNS or psychotropic) and c) medications with indications of mental health. Polypharmacy (of any type), analgesics and prescription medication with a pain-related indication could not be investigated due to low numbers in the uninjured group; these results are presented descriptively only. Parameters were estimated via maximum likelihood. Incidence Rate Ratios (IRR) are presented. All models were bootstrapped with 1,000 replications and report bias-corrected 95% Confidence Intervals (CI) and compared the overall injured group, those injured with/without associated limb loss and those injured with/without deployment-related TBI to the uninjured group. Age at follow up assessment, socioeconomic status and time since injury were added as confounders/covariates to all models.

All medications reported during the clinical interview were coded, and individuals without any medication data reported during the clinical interview were presumed to not be on a prescription medication. Indication was not present for medications reported by one participant and not coded due to insufficient details for one participant. Due to low levels of missing data, this was handled using casewise deletion. One participant experienced a serious injury outside of military service and another participant disclosed a health condition that was present prior to sampled deployment in line with exclusion criteria for the ADVANCE cohort study. These participants were excluded from analyses.

## 3 Results

This analysis included all participants who attended the follow up assessment of ADVANCE (n = 1,051). The uninjured group included n = 525 participants and the injured group n = 526, which comprised of n = 141 individuals injured with associated limb loss, as well as n = 226 individuals injured with deployment-related TBI. Participants were a median age of 37 years old (interquartile range 34, 41) at first follow up assessment (Table 1). Participants were predominantly junior rank at sampling (72.0%), and of White ethnicity (90.4%). Injured personnel had a median new injury severity score of 12 (interquartile range 5, 22).

**TABLE 1:** Demographic results stratified by injury status and prescription medication.

​	Uninjured group		All combat injuries		Limb loss injuries				Deployment-related TBI			
Demographics	Overall uninjured group n = 525 n (% (95%CI))		Overall injured group n = 526 n (% (95%CI))		Injured with limb loss n = 141 n (% (95%CI))		Injured without limb loss n = 385 n (% (95%CI))		Injured with deployment-related TBI n (% (95%CI))		Injured without deployment-related TBI n (% (95%CI))	
​	No medication N = 429	Prescription medication N = 96	No medication N = 368	Prescription medication N = 158	No medication N = 93	Prescription medication N = 48	No medication N = 275	Prescription medication N = 110	No medication N = 138	Prescription medication N = 88	No medication N = 230	Prescription medication N = 70
Median age at follow-up assessment in years (IQR)	38 (34, 41)	39 (35, 43)	37 (34, 41)	38 (35, 42)	36 (33, 40)	37 (35, 41)	37 (34, 41)	38 (35, 43)	37 (34, 41.5)	37 (34, 41.5)	37 (33, 40)	38 (34, 42)
Time from sampling to follow-up assessment* in years, median (IQR)	11 (10, 13)	11 (10, 13)	12 (10, 13)	12 (10, 13)	11 (10, 12)	11 (10, 12)	12 (10, 13)	12 (10, 14)	12 (10, 13)	12 (10, 13)	12 (10, 13)	12 (11, 13)
Ethnicity *n* (%)	​	​	​	​	​	​	​	​	​	​	​	​
Ethnic minority (excluding white minorities)	36 (8.8%)	12 (11.6%)	36 (9.7%)	15 (10.4%)	8 (8.2%)	5 (11.8%)	28 (10.1%)	10 (9.8%)	13 (9.3%)	7 (8.5%)	23 (9.9%)	8 (12.8%)
Rank at sampling *n* (%)	​	​	​	​	​	​	​	​	​	​	​	​
Junior non-commissioned officer/other rank	253 (67.0%)	56 (65.6%)	259 (77.2%)	109 (74.8%)	78 (89.2%)	34 (76.8%)	181 (73.4%)	75 (74.0%)	92 (73.7%)	66 (80.6%)	167 (79.2%)	43 (67.3%)
Senior non-commissioned officer	112 (23.9%)	30 (28.0%)	63 (15.4%)	37 (20.9%)	7 (5.8%)	∼	56 (18.5%)	27 (21.9%)	29 (19.0%)	16 (15.8%)	34 (13.4%)	21 (2.8%)
Officer	64 (9.1%)	10 (6.4%)	46 (7.4%)	12 (4.3%)	8 (5.0%)	∼	38 (8.1%)	8 (4.1%)	17 (7.3%)	6 (3.6%)	29 (7.4%)	6 (5.1%)
Median new injury severity score (IQR)	NA	NA	12 (5, 21.5)	14 (6, 27)	22 (14, 29)	24 (17.5, 35)	9 (4, 13)	11 (4, 22)	12 (6, 27)	22 (9, 29)	11 (4, 17)	12 (6, 22)
IQR interquartile range. NA Not Applicable. Weighted percentages and confidence intervals are presented, along with unweighted cell counts. *Calculated from the difference between age at index injury for injured personnel or age at sampled deployment+0.5 (to reflect average age during sampled year) for the uninjured group and age at baseline/follow-up assessment. ∼ Some data suppressed to allow for confidentiality in line with Defence Statistics rounding policy (https://www.gov.uk/government/publications/defence-statistics-policies/ministry-of-defence-disclosure-control-and-rounding-policy).												

### 3.1 Prevalence of prescription medication use

Prescription medication use was reported by 17.8% (n = 96) participants in the uninjured group and 29.7% (n = 158) in the injured group (Figure 1). Of those on a prescription medication, the median number of prescription medications was 1 (interquartile range 1, 2) in both the injured and uninjured groups. In the uninjured group, 8.0% (n = 41) reported use of CNS medications, 6.3% (n = 32) psychotropic medications and 2.2% (n = 12) analgesic medications. In the injured group, 17.2% (n = 88) reported use of CNS medications, 11.8% (n = 60) psychotropic medications, and 8.7% (n = 45) analgesic medications. Prescription medications with an indication for a mental health condition were reported by 6.0% (n = 30) participants in the uninjured group and 9.6% (n = 48) participants in the injured group, while medications with a pain indication were reported by 2.1% (n = 12) participants in the uninjured group and 9.1% (n = 48) in the injured group. The two most reported prescription medications were the same in both the uninjured and injured groups; sertraline (uninjured n = 9; injured group n = 19) and omeprazole (uninjured group n = 8; injured group n = 17).

**FIGURE 1:**
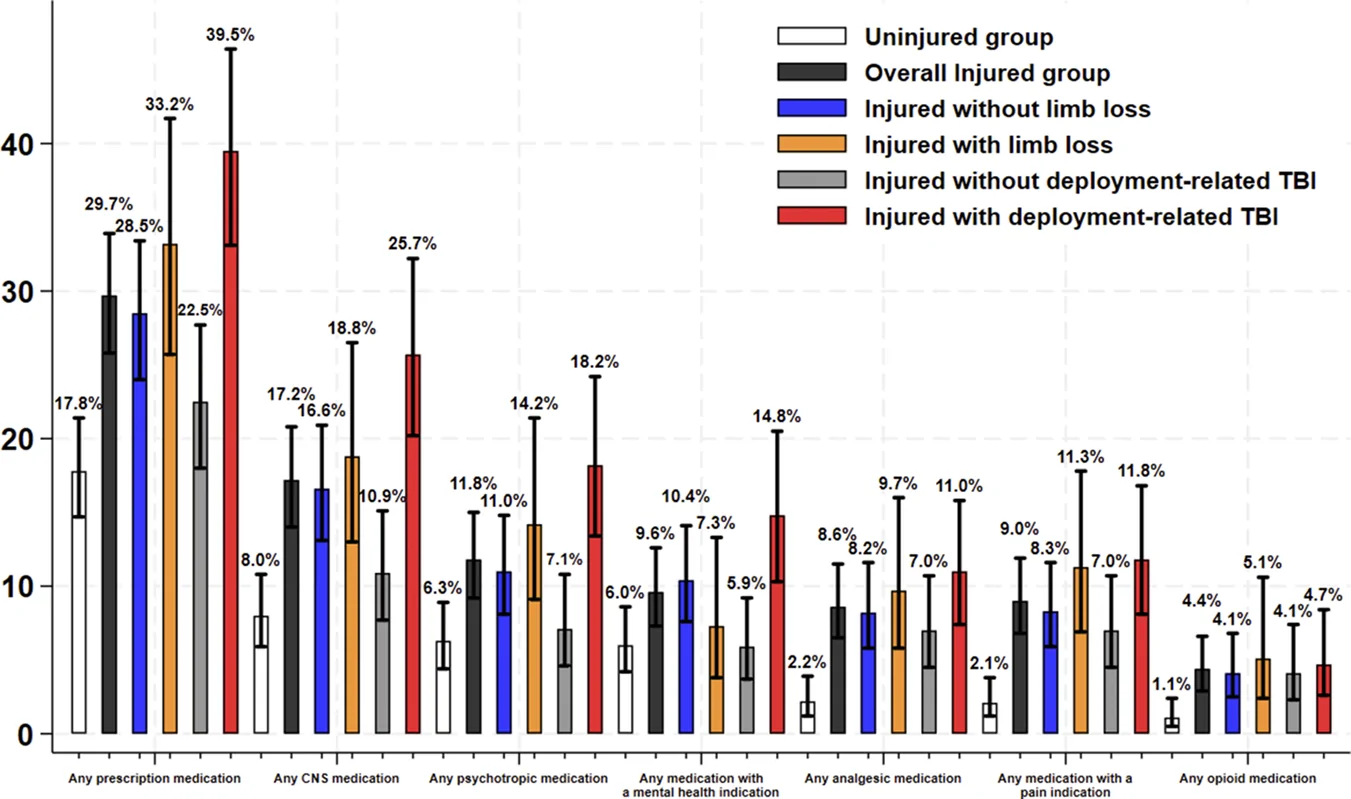
Weighted rates and 95% confidence intervals of prescription medication use amongst seriously injured personnel, injury subgroups and the uninjured group.

The use of opioid analgesics was reported in 1.1% (n = 7) of the uninjured group and 4.4% (n = 22) of the injured group (Figure 1; Supplementary Material 1). Use of gabapentin was reported in n < 5 (data not shown) of the uninjured group and 4.4% (n = 23) of the injured group. Use of pregabalin was reported in n < 5 (data not shown) of the uninjured group and 2.8% (n = 23) of the injured group. Opioid use alongside use of gabapentin/pregabalin was reported by n = 0 participants in the uninjured group and 1.1% (n = 6) participants in the injured group.

The prevalence of personnel who reported use of psychotropic with/without mental health indications and analgesics with/without pain indications were also investigated (Supplementary Materials 2). Psychotropic medications were prescribed without a reported mental health-related indication for 18.4% (n = 7/32) of the uninjured group and 39.5% (n = 24/60) of the injured group. Among participants with limb loss who were prescribed a psychotropic medication, the majority (64.6%; n = 12/19) reported non–mental health indications (e.g., sleep or pain). This proportion was lower among participants with deployment-related TBI (40.6%; n = 17/38).

### 3.2 Prevalence of polypharmacy

General polypharmacy was reported in n < 5 (data not shown) of the uninjured group and 2.6% (n = 15) of the injured group (Figure 2). Psychotropic polypharmacy was reported by 0.9% (n = 5) participants in the uninjured group and 2.4% (n = 12) in the injured group. Analgesic polypharmacy was reported by n < 5 (data not shown) participants in the uninjured group and 1.9% (n = 10) in the injured group. CNS polypharmacy could not be reported due to very low numbers.

**FIGURE 2:**
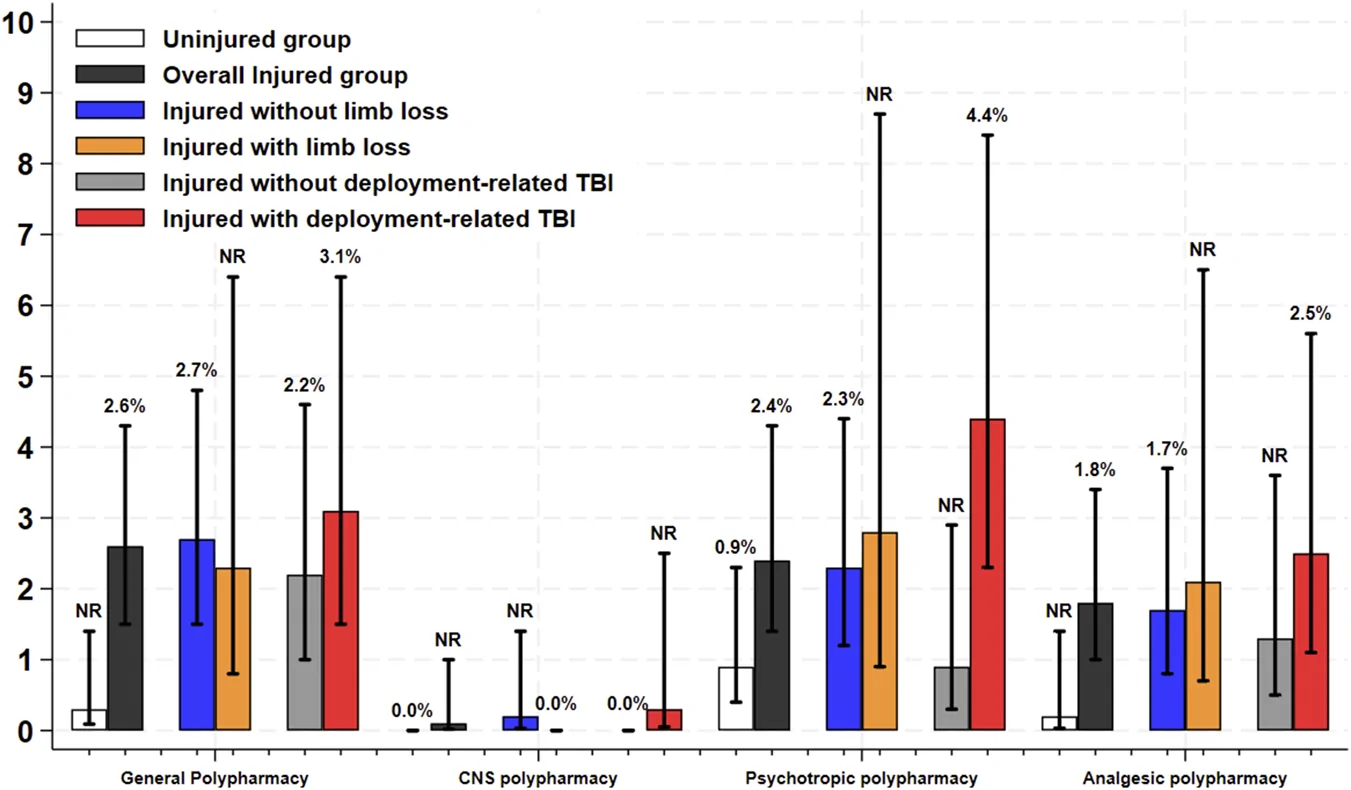
Weighted rates and 95% confidence intervals of polypharmacy amongst seriously injured personnel, injury subgroups and the uninjured group.

### 3.3 Physical combat injuries

Personnel who sustained a physical combat injury were currently using on average more than double the number of prescription medications observed in the uninjured group (IRR 2.15 (1.66, 2.92)) (Figure 3). Elevated counts were also observed for prescriptions of medications acting on the CNS (IRR 2.67 (1.77, 4.08)) and psychotropic medications (IRR 2.03 (1.27, 3.25)). The average number of prescriptions for medications with a mental health indication (IRR 1.54 (0.97, 2.55)) were elevated, but do not rule out no effect.

**FIGURE 3:**
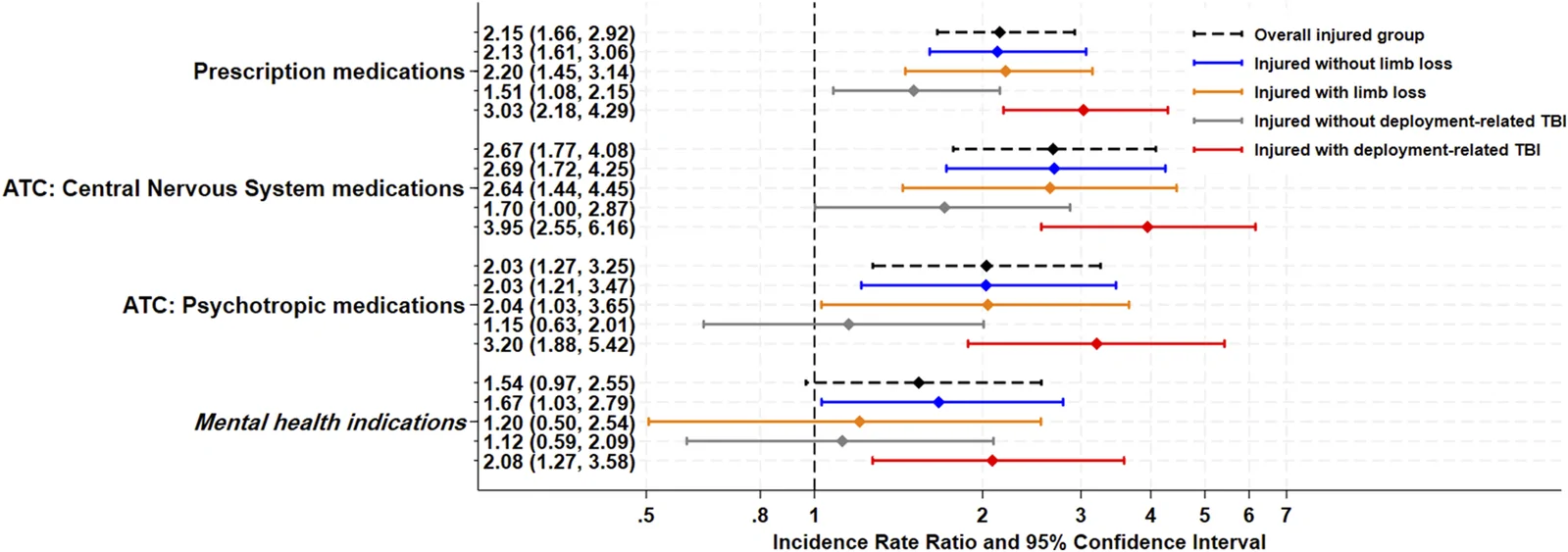
Incidence rate ratios from negative binomial generalised linear model investigating combat injury and rates of prescription medication use, compared to the uninjured group.

### 3.4 Physical combat injuries without limb loss

Personnel who sustained an injury without associated limb loss were currently using on average more than double the number of prescription medications observed in the uninjured group (IRR 2.13 (2.13 (1.61, 3.06)) (Figure 3). Similar elevated counts were observed for prescriptions of medications acting on the CNS (IRR 2.69 (1.72, 4.25)) and psychotropic medications (IRR 2.03 (1.21, 3.47)). The average number of prescriptions for medications with a mental health indication (IRR 1.67 (1.03, 2.79)) were also elevated.

### 3.5 Physical combat injuries with limb loss

Personnel who sustained an injury with associated limb loss were currently using on average more than double the number of prescription medications observed in the uninjured group (IRR 2.20 (1.45, 3.14)) (Figure 3). Elevated counts were also observed for prescriptions acting on the CNS (IRR 2.64 (1.44, 4.45)) and psychotropic medications (IRR 2.04 (1.03, 3.65)). The average number of prescriptions for medications with a mental health indication was slightly higher (IRR 1.20 (0.50, 2.54)), however the small effect size and wide confidence intervals provide no strong evidence for a difference in prescribing practices compared to the uninjured group.

### 3.6 Physical combat injury without deployment-related traumatic brain injury

Compared to the uninjured group, personnel who sustained a physical combat injury without deployment-related TBI were currently using on average 51% more prescription medications (IRR 1.51 (1.08, 2.15)) and 70% more medications acting on the CNS (IRR 1.70 (1.00, 2.87)) (Figure 3). Very small elevations in the average count of prescribed psychotropic medications (IRR 1.15 (0.63, 2.01)) and medications with a mental health indication (IRR 1.12 (0.59, 2.09)) alongside wide confidence intervals suggest no strong evidence for a difference in prescribing practice compared to the uninjured group.

### 3.7 Physical combat injury with deployment-related traumatic brain injury

Personnel who sustained a physical combat injury with deployment-related TBI were currently using on average triple the number of prescription medications observed in the uninjured group (IRR 3.03 (2.18, 4.29)) (Figure 3). Elevated counts were also observed for prescription medications acting on the CNS (IRR 3.95 (2.55, 6.16)), psychotropic medications (IRR 3.20 (1.88, 5.42)) and medications with a mental health indication (IRR 2.08 (1.27, 3.58)).

## 4 Discussion

In this study, we aimed to investigate prescription medication use and polypharmacy in a group of UK Armed Forces personnel who sustained physical combat injuries (injured group) and compare them to a demographically similar group of UK Armed Forces personnel who sustained no such injuries (uninjured group). 17.8% of the uninjured group and 29.7% of the injured group were on at least one prescription medication, with the highest prevalence observed in those who experienced deployment-related TBI (39.5%). We found that the injured group, including those injured with and without limb loss, were currently using twice as many prescription medications as that observed in the uninjured group, and three times as many among those with deployment-related TBI. Similar patterns were observed for CNS, and psychotropic medications. However, the average count of prescriptions with a mental health indication were similar between those injured with limb loss and the uninjured group, demonstrating that psychotropic medications were being prescribed predominantly for non-mental health indications. Overall, polypharmacy was uncommon in this cohort, although greater prevalence was observed in the injured group compared to the uninjured group.

Previous research has explored pain and mental health outcomes in this cohort, observing a greater likelihood of reporting probable generalised anxiety, depression, PTSD and pain in the injured group compared to the uninjured group (Dyball et al., 2022; Vollert et al., 2024; Dyball et al., 2026). Personnel injured with associated limb loss had similar likelihood of reporting probable mental illness and pain compared to the uninjured group, whilst those injured without limb loss or with deployment-related TBI had elevated likelihood of reporting these outcomes. In this study, we see that the use of prescription psychotropics was higher amongst those injured with associated limb loss (14.2%) compared to the uninjured group (6.3%), but not for mental health indications (7.3% and 6.0% respectively). Instead, those injured with limb loss appear to be on psychotropic medications for predominantly other indications, e.g., pain or sleep (Supplementary Materials 2). Whilst it may be possible that psychotropic medication use may provide a protective effect against future psychiatric outcomes, possibly contributing to the wellbeing of those injured with limb loss, this does not appear to be the case for those with deployment-related TBI, who we have observed have higher risk of reporting probable mental illness (Dyball et al., 2022; Blissett et al., 2026; Dyball et al., 2026). This topic remains difficult to investigate prospectively. For example, non-psychiatric indications that may utilise psychotropic medications are often themselves risk factors for mental illness (Gentry et al., 2025; Roughan et al., 2021), and the role of psychotropic medications in pain regulation is complex.

The effects of psychotropics in pain are thought to be mechanistically related to the noradrenergic, inflammatory and serotonergic effects of the medications (Gentry et al., 2025; Sutherland et al., 2018), however little research is available to confirm these mechanisms (Belinskaia et al., 2019). Non-opioid psychiatric medications have been shown to be associated with short-term benefits to pain, but not in the long-term (Ayub et al., 2024). In a recent review on the use of antidepressants for pain management in chronic pain, it was found that the use of antidepressants had a small to moderate effect on mood, but were generally not reliable at eliciting substantial pain relief (Birkinshaw et al., 2024), though it is possible that particular pain phenotypes (e.g., neuropathic) may be responsive to psychotropic medications (Soliman et al., 2025). This is further complicated by the presence of phantom pain, which has limited/conflicting evidence for the effectiveness of antidepressive agents (Culp and Abdi, 2022). Phantom limb pain has been reported of at least moderate intensity in 32% of those injured with limb loss in the ADVANCE cohort (Vollert et al., 2024), yet only 9.7% report use of any analgesics and 11.2% medications with a pain indication (5.1% opioids; 5.5% gabapentin/pregabalin) (Figure 1; Supplementary Material 1). This could represent the recognised limited efficacy of gabapentinoids in neuropathic pain due to side effects such as sedation, or indicate that for persistent pain, non-pharmacological strategies are being employed to limit impact on quality of life (Soliman et al., 2025), though further research would be required to confirm this.

The prevalence of prescription medication use was relatively low (17.8%–39.6%) compared to the UK general population rates, with the Health Survey for England estimating that up to 48% of adults being on a prescription medication (NHS Digital, 2016). The prevalence of polypharmacy (0–4.4%) and opioid use (1.1%–5.1%) was also relatively low in this cohort, with opioid pain medications estimated to be used by 13% of adults in England (Public Health England Prescribed medicines review: summary, 2020). Whilst the lower prevalence of prescribing compared to the general population is reassuring, no known UK estimates of polypharmacy relevant to this population (relatively young male combat injured UK Armed Forces personnel) could be found, and it is of note that direct comparisons with the UK general population are limited by important demographic and health differences, such as inclusion of older individuals, women, chronic disease and a wider range of disabilities that might affect prescription medication use. Prevalence of opioid use in Iraq/Afghanistan US veterans utilising Veterans Health Administration care has been estimated to be 23%, increasing to 35% for those with a diagnosis of TBI (Toblin et al., 2014), though once again differences in prescribing practices of opioids between these countries should be considered when comparing UK and US prevalence rates (Weisberg et al., 2014).

Gabapentinoids continue to be recommended as a first line agents for neuropathic pain (Soliman et al., 2025), though there is increasing concern about the risks of long-term use including risk of drug misuse and overdose (Muller et al., 2024). Rates of gabapentin and pregabalin prescribing have increased over the last decade in the UK but in 2019, they became Schedule three drugs, with specific restrictions on prescribing (Goodman and Brett, 2017; Chan et al., 2023). Safety alerts have been issued about the risk of respiratory depression when co-prescribed with opioid medication (UK Home Office Control of pregabalin and gabapentin under the misuse of drugs act 1971, 2019). The very low prevalence of co-prescription in this cohort is therefore encouraging. Prevalence of opioid use disorder (OUD) is high in combat-injured US veteran populations (Kelley et al., 2019). Although this analysis solely focuses on prescribed medication, exposure to prescribed opioids is a known risk factor for OUD, especially amongst those with chronic pain (Bennett et al., 2013). In the 2010s, studies demonstrated that veterans receiving opioid prescriptions for pain in the US had nearly twice the risk of fatal accidental poisoning compared to the general population (Bohnert et al., 2011). This led to the introduction of a series of measures to improve opioid stewardship and the publication of clinical guidelines advising against the use of long-term opioids for chronic pain (US Veterans Health Administration, 2024). As a result, rates of opioid prescribing have decreased (Sandbrink et al., 2020). The low rates of opioid prescribing in this British cohort suggest adherence to UK clinical guidelines (National Instutite for Health and Care Excellence, 2025).

This paper has important clinical implications. Whilst some medication-related risk has been observed in military populations previously, e.g., opioids, clinicians may not be aware of the risks that polypharmacy poses in young to middle aged adults, and greater consideration of the risks associated with the use of multiple medications is warranted. In the context of polytrauma, guidelines for medication use to target pain and mental health outcomes within strata of injuries (e.g., TBI, amputation) can differ, often without consideration of additional comorbidity making prescribing practices siloed (Brown-Taylor et al., 2021). There is an increasing network of clinicians with specialist knowledge and expertise in veteran care in the UK, through initiatives such as Op VALOUR, Op RESTORE and Op COURAGE and veteran friendly accreditation in primary care. Such networks would be well placed to collaboratively develop strategies to mitigate and highlight risks associated with polytrauma and polypharmacy. This is especially relevant as whilst the Beers criteria exists for older adults (Fick et al., 2003), no such criteria currently exists for assessing polypharmacy in young-middle aged adults. We recommend that a Delphi consensus process be undertaken to develop guidance on prescription medication use in younger individuals with complex, competing physical comorbidities/polytrauma.

It is important to note that this cohort consists of relatively young men (median age 37 years at time of assessment) who, at the time of injury, had high levels of physical fitness and underwent an extended period of rehabilitation post-injury at the UK Defence Medical Rehabilitation Centre to achieve the highest level of functioning possible. The use of analgesic medications in this group may be necessary for the patient’s quality of life in the case of complex polytrauma, however risks to health when using these medications long-term should not be ignored (Li et al., 2022; Guillot et al., 2024). Continued observation of this cohort is vital to understand the risks that develop with this type of pharmacological treatment as age-related conditions become more common and polypharmacy occurs more often. Future research will also explore the non-pharmacological treatments being undertaken by the cohort and attempt to understand the factors associated with good pain management following combat injury.

This study benefits from the use of a robust statistical approach, controlling for *a-priori* confounders and utilising a large, detailed dataset of medications from a representative group of UK Armed Forces personnel who sustained physical combat injuries during the conflict in Afghanistan and a well-matched group of demographically similar personnel who sustained no such injuries. However, this study also has several limitations. Whilst participants were encouraged to bring their medications to the appointment day, some did not, and may have misremembered or not reported details, with some medications possibly not reported due to social desirability bias. If individuals failed to report these medications, they would have been incorrectly labelled as not on a medication and increase the risk of type two error. Other studies have investigated concordance between self-reported medications and medical records, and found that for long-term medication use there is generally moderate-high agreement between these methods (Sediq et al., 2018). Investigation of other details of prescription medication use, such as route, dose or frequency, were beyond the scope of the current study, but investigation may yield useful clinical data in the future. Due to large heterogeneity in polytrauma, it is difficult to classify injured personnel beyond broad categories (e.g., with/without limb loss or TBI). Groups were also not mutually exclusive, so individuals in the limb loss group may also have been in the TBI group, and combinations could not be investigated due to small sample sizes. Similarly, Mayo classifications of TBI (e.g., mild, moderate-severe) could not be investigated due to small sample sizes. Mental health and pain are potential mediators/on the causal pathway between combat injury and medication use. These factors were not investigated as a mediation analysis was outside of the scope of this paper. The Polytrauma Clinical Triad, a pattern of post-traumatic stress disorder, TBI and chronic pain that has been identified as being prevalent in US veterans who deployed to Afghanistan, may be of particular interest, and future studies in the ADVANCE cohort may investigate this phenomenon in the context of prescription medication practices and polypharmacy (Brown-Taylor et al., 2021).

## 5 Conclusion

UK Armed Forces personnel who sustained physical combat injuries during deployment to Afghanistan were using more than twice as many prescription medications as demographically similar personnel without such injuries. This elevated use of prescription medications was observed in both those with and without limb loss, with the highest levels among individuals with deployment-related TBI. Despite this increased level of prescribing, the prevalence of general, CNS, psychotropic, and analgesic polypharmacy remained low across the cohort.

## Data Availability

The datasets presented in this article are not readily available because of the sensitive nature of the data collected for ADVANCE, its data cannot be made publicly available, but requests to access the dataset from qualified researchers trained in human subject confidentiality may be sent to adv_data_team@imperial.ac.uk and will be considered by the Project Board on a case-by-case basis, and subject to UK Ministry of Defence clearance. Details of the study data are available on the ADVANCE website (www.advancestudydmrc.org.uk/researchportal). Requests to access the datasets should be directed to adv_data_team@imperial.ac.uk.

## In memoriam

This paper is dedicated to the memory of Professor Nicola T Fear who sadly passed away during the writing of this manuscript. She leaves behind a legacy of research that will positively impact the wellbeing of the Armed Forces community for generations to come. We all loved you and miss you dearly.
