# Supplementary material for: Self-reported prescription medication use and polypharmacy following physical combat injury in the UK: the ADVANCE cohort study
Source: Front Pharmacol. 2026 Oct 1;17:1893711. doi: 10.3389/fphar.2026.1893711 (PMC13670212; doi:10.3389/fphar.2026.1893711)
Supplement: Supplementary file 1 [file Table1.docx]

Supplementary materials 1: Prescription medications and general polypharmacy, central nervous system polypharmacy, psychotropic polypharmacy and pain polypharmacy amongst injured personnel and uninjured group personnel

|  | Overall Uninjured group n=525  n (% (95%CI)) | Overall injured group n=526  n (% (95%CI)) | Injured with limb loss n=141  n (% (95%CI)) | Injured without limb loss n=385  n (% (95%CI)) | Injured with deployment-related TBI  n (% (95%CI)) | Injured without deployment-related TBI  n (% (95%CI)) |
| --- | --- | --- | --- | --- | --- | --- |
| Any prescription medication | 96 (17.8 (14.7, 21.4)) | 158 (29.7 (25.8, 33.9)) | 48 (33.2 (25.7, 41.7)) | 110 (28.5 (24.0, 33.4)) | 88 (39.5 (33.1, 46.4)) | 70 (22.5 (18.0, 27.7)) |
| *1-4* | ~ | 143 (27.1 (23.3, 31.2)) | ~ | 99 (25.8 (21.5, 30.6)) | 80 (36.4 (30.1, 43.2)) | 63 (20.3 (16.0, 25.4)) |
| *General polypharmacy (5+) (range 5-9)* | ~ | 15 (2.6 (1.5, 4.3) | ~ | 11 (2.7 (1.5, 4.8)) | 8 (3.2 (1.5, 6.4)) | 7 (2.2 (1.0, 4.6)) |
| **Anatomical Therapeutic Classification: System classifications** | | | | | | |
| Any Central Nervous System medications | 41 (8.0 (5.9, 10.8)) | 88 (17.2 (14.0, 20.8)) | 26 (18.8 (13.0, 26.5)) | 62 (16.6 (13.1, 20.9)) | 55 (25.8 (20.2, 32.3)) | 33 (10.9 (7.7, 15.1)) |
| *1-4* | 41 (8.0 (5.9, 10.8)) | ~ | 26 (18.8 (13.0, 26.5)) | ~ | ~ | 33 (10.9 (7.7, 15.1)) |
| *CNS polypharmacy (5+) (range( ~)* | 0 (0 (0, 0)) | ~ | 0 (0 (0, 0)) | ~ | ~ | 0 (0 (0, 0)) |
| **Anatomical Therapeutic Classification: Therapeutic classifications** | | | | | | |
| Any psychotropic medications | 32 (6.3 (4.4, 8.9)) | 60 (11.8 (9.2, 15.0)) | 19 (14.2 (9.1, 21.4)) | 41 (11.0 (8.1, 14.8)) | 38 (18.2 (13.4, 24.2)) | 22 (7.1 (4.6, 10.8)) |
| *1* | 27 (5.4 (3.7, 7.8)) | 48 (9.4 (7.1, 12.4)) | ~ | 32 (8.8 (6.2, 12.3)) | 29 (13.8 (9.6, 19.3)) | ~ |
| *Psychotropic polypharmacy (2+) (range 2-2)* | 5 (0.9 (0.4, 2.3)) | 12 (2.4 (1.4, 4.3)) | ~ | 9 (2.3 (1.2, 4.4)) | 9 (4.4 (2.3, 8.4)) | ~ |
| Any analgesic medications | 12 (2.2 (1.2, 3.9)) | 45 (8.6 (6.5, 11.5)) | 14 (9.8 (5.8, 16.0)) | 31 (8.2 (5.8, 11.6)) | 25 (10.9 (7.4, 15.8)) | 20 (7.0 (4.5, 10.7)) |
| *1* | ~ | 35 (6.8 (4.9, 9.4)) | ~ | 24 (6.5 (4.3, 9.6)) | 19 (8.4 (5.4, 12.9)) | ~ |
| *Analgesic polypharmacy (2+) (range 2-5)* | ~ | 10 (1.8 (1.0, 3.4)) | ~ | 7 (1.7 (0.8, 3.7)) | 6 (2.5 (1.1, 5.6)) | ~ |
| Any opioid medication | 7 (1.1 (0.5, 2.4)) | 22 (4.4 (2.9, 6.6)) | 7 (5.1 (2.4, 10.6)) | 15 (4.1 (2.5, 6.8)) | 11 (4.7 (2.6, 8.4)) | 11 (4.1 (2.3, 7.4)) |
| Any Gabapentin/Pregabalin medication | ~ | 23 (4.3 (2.9, 6.5)) | 8 (5.5 (2.7, 10.8)) | 15 (3.9 (2.3, 6.5)) | 15 (6.6 (4.0, 10.8)) | 8 (2.6 (1.3, 5.4)) |
| **Self-report indications** | | | | | | |
| Medication with mental health indication | 30 (6.0 (4.2, 8.6)) | 48 (9.6 (7.2, 12.6)) | 10 (7.3 (3.8, 13.3)) | 38 (10.4 (7.6, 14.1)) | 29 (14.7 (10.3, 20.5)) | 19 (5.9 (3.7, 9.2)) |
| Medication with pain indication | 12 (2.1 (1.2, 3.8)) | 48 (9.0 (6.8, 11.9)) | 16 (11.3 (6.9, 17.8)) | 32 (8.3 (5.9, 11.6)) | 27 (11.8 (8.1, 16.8)) | 21 (7.0 (4.5, 10.7)) |
| Weighted percentages and confidence intervals are presented.  CI Confidence Interval  ~ Some data suppressed to allow for confidentiality in line with Defence Statistics rounding policy (https://www.gov.uk/government/publications/defence-statistics-policies/ministry-of-defence-disclosure-control-and-rounding-policy). | | | | | | |

Supplementary materials 2: Therapeutic classes stratified by mental health/pain indications

|  | Overall Uninjured group n=525  n (% (95%CI)) | Overall injured group n=526  n (% (95%CI)) | Injured with limb loss n=141  n (% (95%CI)) | Injured without limb loss n=385  n (% (95%CI)) | Injured with deployment-related TBI n=226  n (% (95%CI)) | Injured without deployment-related TBI n=300  n (% (95%CI)) |
| --- | --- | --- | --- | --- | --- | --- |
| Psychotropic medication | 32 (6.3 (4.4, 8.9)) | 60 (11.8 (9.2, 15.0)) | 19 (14.2 (9.1, 21.4)) | 41 (11.0 (8.1, 14.8)) | 38 (18.2 (13.4, 24.2)) | 22 (7.1 (4.6, 10.8)) |
| Psychotropic medication without mental health indication | 7/32 (18.5 (8.3, 36.0)) | 24/60 (39.5 (27.3, 53.1)) | 12/19 (64.7 (39.0, 84.0)) | 12/41 (28.7 (16.2, 45.6)) | 17/38 (40.6 (25.5, 57.6) | 7/22 (37.4 (17.9, 62.0)) |
| Non-mental health Indications reported | Sleep n=3 Pain n=3 Other n=1 | Sleep n=10 Pain n=10 Other n=3 Missing n=1 | Sleep n=7 Pain n=5 | Sleep n=3 Pain n=5 Other n=3 Missing n=1 | Sleep n=8  Pain n=5  Other n=3 Missing n=1 | *Sleep n=2 Pain n=5* |
| Analgesic medication | 12 (2.2 (1.2, 3.9)) | 45 (8.6 (6.5, 11.5)) | 14 (9.8 (5.8, 16.0)) | 31 (8.2 (5.8, 11.6)) | 25 (10.9 (7.4, 15.8)) | 20 (7.0 (4.5, 10.7)) |
| Analgesic medication without pain indication | ~ | 5 (11.4 (4.5, 26.1)) | ~ | ~ | ~ | ~ |
| Non-pain indications reported | ~ | Mental health n=4  Missing n=1 | ~ | ~ | ~ | ~ |
| Weighted percentages and confidence intervals are presented alongside unweighted cell counts. | | | | | | |
